# Reducing Wait Time and Enhancing Care: Outcomes of a Pharmacist Prescriber Clinic in Perinatal Psychiatry

**DOI:** 10.1192/bjo.2026.11545

**Published:** 2026-06-30

**Authors:** Katie Burton, Deepa Bagepalli Krishnan

**Affiliations:** 1 Nottinghamshire Healthcare NHS Foundation Trust, Nottingham, United Kingdom; 2 University of Nottingham, Nottingham, United Kingdom

## Abstract

**Aims:**

A prescribing model was co-developed with consultant psychiatrists and pharmacist peers to establish the pilot clinic in June 2024. The pharmacy prescriber clinic was set up with an aim to reduce appointment wait times and improve efficiency. This study evaluated the pilot pharmacist prescriber clinic within a perinatal community mental health team from June 2024 to June 2025 using continuously collected quantitative and qualitative data. Patients and relatives attending face-to-face appointments were invited to complete anonymous feedback forms. The pharmacist held weekly clinics, providing pre-conception and follow-up appointments, managing urgent patient calls, and responding to queries from general practitioners (GPs), local mental health teams (LMHTs), consultants, and perinatal community psychiatric nurses (PCPNs). Clinical supervision was provided after each clinic by a perinatal consultant psychiatrist.

**Methods:**

A quality improvement approach was used through the evaluation of the problem (longer wait times), stakeholder engagement, and continuous measurement in the planning and design of the pilot clinic. Data was collected on all consultations and interventions. Appointment wait times were the main outcome measure. Feedback was sought from patients, relatives, and perinatal colleagues.

**Results:**

Pre-conception wait times decreased from an average of 43.7 days (6.2 weeks) to 15.6 days (2.2 weeks). Across the study period, the pharmacist conducted 86 interventions: 20 pre-conception consultations, 10 follow-ups, 5 patient calls, 11 GP queries, 3 LMHT queries, 33 consultant/resident doctor queries, 3 PCPN queries, and 1 referral query. A total of 17 feedback forms were completed (11 patients, 5 partners, 1 supporter). Respondents unanimously agreed they were treated with dignity and respect, received the information they required, and found the pharmacist polite and approachable. Sixteen rated the consultation “very helpful”, and one rated it “quite helpful.” All rated the service as excellent. All respondents strongly agreed that they were provided with all the information they wanted and felt they needed. Colleague feedback highlighted that the clinic shortened wait times, improved consultant capacity to manage other complex issues, improved patient care and safety, provided comprehensive and valued information, and facilitated robust care planning. Consultants also reported that pharmacist clinic letters served as exemplars for training resident doctors and non-medical prescribers.

**Conclusion:**

The pharmacist-prescriber clinic significantly reduced pre-conception wait times, released consultant time, and enhanced both patient care and safety. Initially launched as a pilot, the clinic has continued due to its demonstrable benefits, although future fundingarrangements remain under discussion.

